# Multi-Omics and Targeted Approaches to Determine the Role of Cellular Proteases in *Streptomyces* Protein Secretion

**DOI:** 10.3389/fmicb.2018.01174

**Published:** 2018-06-04

**Authors:** Tobias Busche, Konstantinos C. Tsolis, Joachim Koepff, Yuriy Rebets, Christian Rückert, Mohamed B. Hamed, Arne Bleidt, Wolfgang Wiechert, Mariia Lopatniuk, Ahmed Yousra, Jozef Anné, Spyridoula Karamanou, Marco Oldiges, Jörn Kalinowski, Andriy Luzhetskyy, Anastassios Economou

**Affiliations:** ^1^Center for Biotechnology, Bielefeld University, Bielefeld, Germany; ^2^Institute for Biology-Microbiology, Freie Universität Berlin, Berlin, Germany; ^3^Department of Microbiology and Immunology, Rega Institute, KU Leuven, Leuven, Belgium; ^4^IBG-1: Biotechnology, Forschungszentrum Jülich GmbH, Institute of Bio- and Geosciences, Jülich, Germany; ^5^Helmholtz-Zentrum für Infektionsforschung GmbH, Braunschweig, Germany; ^6^Pharmazeutische Biotechnologie, Universität des Saarlandes, Saarbrücken, Germany; ^7^Department of Molecular Biology, National Research Centre, Giza, Egypt; ^8^Institute of Biotechnology, RWTH Aachen University, Aachen, Germany

**Keywords:** proteases, expression levels, mRFP, multi-omics, Streptomyces lividans, protein secretion, RNAseq, FtsH

## Abstract

Gram-positive *Streptomyces* bacteria are profuse secretors of polypeptides using complex, yet unknown mechanisms. Many of their secretory proteins are proteases that play important roles in the acquisition of amino acids from the environment. Other proteases regulate cellular proteostasis. To begin dissecting the possible role of proteases in *Streptomyces* secretion, we applied a multi-omics approach. We probed the role of the 190 proteases of *Streptomyces lividans* strain TK24 in protein secretion in defined media at different stages of growth. Transcriptomics analysis revealed transcripts for 93% of these proteases and identified that 41 of them showed high abundance. Proteomics analysis identified 57 membrane-embedded or secreted proteases with variations in their abundance. We focused on 17 of these proteases and putative inhibitors and generated strains deleted of their genes. These were characterized in terms of their fitness, transcriptome and secretome changes. In addition, we performed a targeted analysis in deletion strains that also carried a secretion competent mRFP. One strain, carrying a deletion of the gene for the regulatory protease FtsH, showed significant global changes in overall transcription and enhanced secretome and secreted mRFP levels. These data provide a first multi-omics effort to characterize the complex regulatory mechanisms of protein secretion in *Streptomyces lividans* and lay the foundations for future rational manipulation of this process.

## Introduction

Microorganisms of the family *Streptomycetaceae* are filamentously growing key players in soil habitats all around the planet, where they vitally contribute as decomposers to recycle organic material (Hopwood, 2007; Barka et al., 2016; Ranjani et al., 2016). To fulfill this duty, *Streptomyces* produce and secrete a large arsenal of extracellular enzymes including proteases, enabling them to exploit complex proteinogenic resources (Chater, 2016).

Extracellular peptide-cleaving enzymes have a dual function in heterologous protein production. While on one hand being necessary for protein folding and essential steps in the secretion machinery (Gilbert et al., 1995; Neef et al., 2017), proteases are also a challenge to industrial protein production (van den Hombergh et al., 1997), due to their undesired capacity to degrade the recombinant product itself. Therefore, reducing the extracellular protease activity by gene deletions is a typical path toward higher protein production titers and yields in other industrial relevant organisms, such as *Aspergillus* (van den Hombergh et al., 1997; Xu et al., 2000), *Bacillus* (Pohl et al., 2013) and in higher eukaryotic systems like insect cell cultures (Gotoh et al., 2001).

*Streptomyces lividans*, a well-investigated member of the *Streptomyces* family, already innately displays a rather low extracellular protease activity in comparison to other related species (Butler et al., 1993; Gilbert et al., 1995; Liu et al., 2013), while at the same time maintaining a highly active secretion machinery (Anné et al., 2017) and being easily manipulated genetically. Therefore, this strain has become an important model organism for heterologous protein production (Gilbert et al., 1995; Anné et al., 2012; Chater, 2016).

In addition to the extracellular environment, proteases play essential roles within the membrane border of the cell. Amongst these functions are the recycling of misfolded proteins, degradation of unused enzymes as well as general housekeeping tasks (Krishnappa et al., 2013). Regulated proteolysis is a post-translational mechanism with a direct influence on the amount of certain proteins (Langklotz et al., 2012). Regulatory proteolysis is accomplished in Gram-negative bacteria by five ATP-dependent proteases: ClpAP, ClpXP, Lon, HslUV and FtsH, and three other proteases: ClpCP, ClpEP and the proteasome (Gur et al., 2011). Deletion of these regulatory proteases may affect the proteome of the cell. For example, deletion of FtsH strongly increases the abundance of ten cytoplasmic and membrane proteins in *Corynebacterium glutamicum* without effect on its growth (Ludke et al., 2007).

Here, we undertook a broad analysis of TK24 cellular proteases using a transcriptomics and secretomics approach to define proteases of potential interest in the regulation of endogenous and heterologous protein secretion. Using expression levels, growth phase-specific synthesis or apparent biochemical properties we narrowed down our focus to 17 protease genes that were subsequently deleted. Eight of the derivative strains were analyzed with respect to their effect on whole secretome (or “exoproteome”) export and in a more targeted approach, the secretion of a mRFP derivative carrying a Sec pathway signal peptide was studied in 14 of the deletion strains. These experiments revealed that the most significant effect was seen when the core regulatory protease FtsH, which is embedded in the plasma membrane (Walker et al., 2007), was removed. FtsH removal led to significant improvement of secretion at the whole secretome level and, also, at the targeted level of a secreted mRFP. A complex network of both transcription and protein level effects might explain this novel role of FtsH in protein secretion.

This study lays the foundation for application of multi-omics tools to the study of several aspects of protein secretion in TK24 and paves the way toward better understanding and rationally redesigning heterologous protein secretion in these bacteria.

## Results

### Analysis and Identification of Highly Transcribed Proteases

*Streptomyces lividans* TK24 contains 190 protease-encoding genes (Supplementary Table S1; SToPSdb; Tsolis et al., 2018)^1^. Of these, 50% are secreted and another 10% are membrane-embedded. In addition, TK24 secretes 3 proteins that act as protease inhibitors. To determine whether proteases play a role in the secretory processes of TK24, we first classified potential proteases based on several parameters, as follows: (I) the presence of a Sec or Tat secretion signal using multiple bioinformatics tools (Tsolis et al., 2018), (II) whether the protein in question belongs to the set of core genes of *S. lividans* TK24, based on the comparison to the core genome of 13 *Streptomyces* species, and (III) the maximal transcript level, determined by RNAseq. For the latter, TK24 was grown under two media regimes (minimal medium supplemented with glucose and minimal medium with glucose supplemented with casamino acids) and cells were harvested at three different growth phases (early and late logarithmic, and stationary).

**FIGURE 1:**
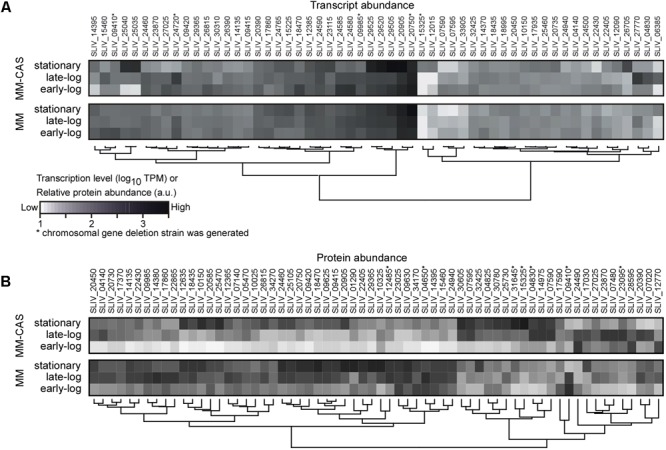
Transcriptomics and secretomics analysis of proteases in TK24. **(A)** Transcript abundance profile for protease encoding genes transcribed highly (TPM > 100) in MM or MM-CAS media for early-log, late-log, and stationary growth phase cultures in at least one condition. Transcript abundance (TPM values) are log-transformed, and grouped by hierarchical clustering using the Wards method and the Euclidean distance between values. Proteases whose genes were deleted (see **Table 1**) are shown with (^∗^). **(B)** Abundance profile for secreted proteases identified by proteomics in MM or MM-CAS media for earl-log, late-log and stationary growth phase cultures. Protein abundance (iBAQ values) are log-transformed, centered, scaled and grouped by hierarchical clustering using the Euclidean distance between values. Proteases whose genes were deleted (see **Table 1**) are shown with (^∗^). *n* = 6–8.

Transcription signals for 93.8% of the annotated encoded proteases could be determined and were used to rank them (**Figure 1A** and Supplementary Table S1). Forty one of the protease-encoding genes were transcribed at high levels, in a growth-phase- and medium-dependent manner. They code for secreted (13), cytoplasmic (18) and membrane-embedded (19) proteases. Some of the membrane-embedded proteases, such as FtsH, are well known proteostatic regulators in other organisms like *Escherichia coli* (Gottesman, 1996). In addition to the proteases, transcription of a gene encoding for a secreted protease inhibitor was detected.

To determine if these transcription signals give rise to detectable secreted protein products, we undertook a proteomics analysis of the secretome of TK24 under the same growth conditions and sampling times along the growth curve of the cell (**Figure 1B**). In total, 82 of the proteases could be detected at the protein level, 57 of them were secreted or membrane-integrated (Supplementary Table S2). A medium correlation of their average transcript and protein abundance was observed between transcriptomics and proteomics experiments (Supplementary Figure S1). In minimal media, the levels of about half of them peaked at early log and late-log phase and while the others were most abundant in the stationary phase. In contrast, when growing in casamino acid-supplemented minimal media, abundance peaks were for most of the proteases at early log or stationary phase. This late phase expression is reminiscent of the secretion from TK24 of some heterologous proteins in various growth media, in which high levels of secretion were linked to the cells entering the stationary phase (Pozidis et al., 2001; Hamed et al., 2017).

In addition to the proteases, secretomics revealed the presence of 3 protease inhibitors including that of subtilisin inhibitor (Uniprot Accession: D6EYB7), commonly seen as the most abundant exported protein in TK24 (Hamed et al., unpublished).

### Selection of Protease Genes of Interest and Creation of a Protease Deletion Library

Given the transcriptomics and the secretomics results, we decided to focus on a small number of proteases and test their role in protein secretion. For this we chose 15 proteases, a protease inhibitor gene and membrane-embedded regulator (**Table 1**). The former included the likely proteostatic regulator FtsH (Langklotz et al., 2012) and its second homologue in *S. lividans* FtsH3 (both membrane-embedded), and Lon, a major proteostatic regulator in *E. coli* (Lee and Suzuki, 2008). We also included 9 secreted proteases. We chose the protease targets based on at least one of the following criteria: high level transcription, possible role in overall regulation, possible direct effect on secreted polypeptides, or secretion patterns that were consistent with early or late appearance in the secretome.

**Table 1:** List of protease and protease inhibitor genes selected for deletion analysis.

No	Name of derivative strain	Deleted gene name	Function	Topology (secretion system)	Structural/Functional domains	Maximal transcription level [TPM]	Secretomics	mRFP secretion	Enzyme Class according to structural domains
1	TK24ΔSLIV_ 09985	SLIV_ 09985	Peptidase S8, subtilisin-related protein	Integral membrane protein (Sec)	IPR015500	639.67		+	Serine protease
2	TK24ΔSLIV_ 20750	SLIV_ 20750	ATP-dependent zinc metalloprotease FtsH (EC 3.4.24.-)	Integral membrane protein (Sec)	IPR005936	2528.62	+	+	Metalloproteases
3	TK24ΔSLIV_ 10535	SLIV_ 10535	ATP-dependent zinc metalloprotease FtsH3	Integral membrane protein (Sec)	IPR005936	6.46	+	+	Metalloproteases
4	TK24ΔSLIV_ 10025	SLIV_ 10025	T7SS peptidase S8A, mycosin-1, component of T7S export system	Integral membrane protein (Sec)	IPR015500	48.02		+	Serine protease
5	TK24ΔSLIV_ 11935	SLIV_ 11935	ATP-dependent serine protease Lon	Cytoplasmic	IPR027065	87.45	+	+	Serine protease
6	TK24ΔSLIV_ 11275	SLIV_ 11275	Neutral zinc metalloprotease	Secreted protein (Sec)	IPR023612	18.57	+	+	Metalloproteases
7	TK24ΔSLIV_ 11270	SLIV_ 11270	Neutral zinc metalloprotease	Secreted protein (TAT)	IPR023612	2.22	+	+	Metalloproteases
8	TK24ΔSLIV_ 15325	SLIV_ 15325	Peptidase, Leupeptin-inactivating enzyme 1	Secreted protein (Sec)	Peptidase_M28 domain IPR007484	121.08		+	
9	TK24ΔSLIV_ 09410	SLIV_ 09410	Peptidase	Secreted protein (Sec)	Peptidase_M23 domain IPR016047	498.44		+	Metalloproteases
10	TK24ΔSLIV_ 24720	SLIV_ 24720	Protein containing Tachylectin 2 domain	Secreted protein (Sec)	IPR023294	733.23		+	
11	TK24ΔSLIV_ 02150	SLIV_ 02150	Extracellular small neutral protease (EC 3.4.24.77)	Secreted protein (Sec)	IPR000013	1.09		+	Metalloproteases
12	TK24ΔSLIV_ 17030	SLIV_ 17030	Peptidase M1, alanine aminopeptidase/leukotriene A4 hydrolase	Secreted protein (Sec)	IPR001930	43.28		+	
13	TK24ΔSLIV_ 31645	SLIV_ 31645	Tripeptidyl aminopeptidase (EC 3.4.14.-)	Secreted protein (Sec)	IPR000073	69.42	+		
14	TK24ΔSLIV_ 12485	SLIV_12485	Peptidase S33 tripeptidyl aminopeptidase-like protein	Secreted Lipoprotein (Sec)	IPR029058	59.60	+		
15	TK24ΔSLIV_ 04650	SLIV_ 04650	Peptidase containing LysM_dom and Peptidase M23 domains	Secreted protein (Sec)	LysM_dom (IPR018392) Peptidase M23 (IPR016047) domains	37.16	+		Metalloproteases
16	TK24ΔSLIV_ 34120	SLIV_ 34120	Probable subtilase-type protease inhibitor	Secreted protein (Sec)	IPR000691	8162.43		+	
17	TK24ΔSLIV_ 28740	SLIV_ 28740	Stomatin family	Integral membrane (Sec)	IPR001972	539.22		+	

*Subcellular topology classification was obtained from STopSdb http://www.stopsdb.eu (Tsolis et al., 2018) using the updated topological annotation of the *Streptomyces lividans* proteome. IPR: Proteins signature based on Integrative proteins signature data base (InterPro database). (+) Represents strains that are included in secretomics analysis or mRFP secretion or both.*

Following the gene selection process, we generated strains with specific single deletions of all the genes of interest, employing an established protocol used extensively for gene deletion experiments (Myronovskyi et al., 2014).

### Fitness- and Production Testing of Strains With Protease Deletions

**FIGURE 2:**
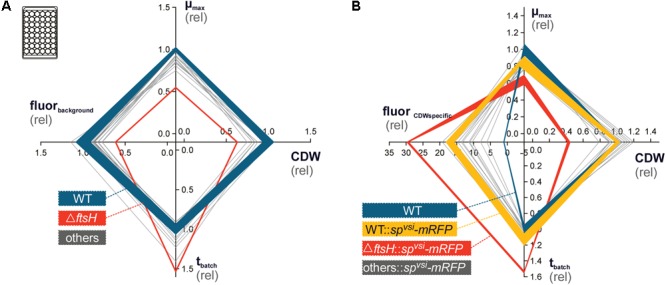
Phenotyping of *S. lividans* protease gene deletion strains, using microtiter-plate cultivation (Koepff et al., 2017). **(A)** Characterization of the protease deletion strain library for growth phenotypes, in advance to recombinant mRFP integration. In total, four parameters μ_max_ (top), cell-dry-weight (CDW, right), cultivation time until stationary phase (t_batch_, bottom) and background RFP fluorescence (fluor_background_, left, ex.: 550 nm, em.: 589 nm) were evaluated. All data was normalized on the wild-type *S. lividans* TK24 (WT, solid blue line), for which the obtained standard deviation (semi-transparent background) is additionally provided. The mutant, exhibiting the strongest phenotype, ΔFtsH, is outlined by a solid red line. **(B)** Phenotyping of the strain library with recombinant mRFP production. Axe labeling is identical to **(A)** except that fluor_background_ was replaced by CDW-specific mRFP fluorescence (fluor_CDWspecific_). Standard deviation is provided for the TK24 (blue) and TK24::*sp^vsi^-mRFP* (yellow), as well as for TK24Δ*ftsH*::*sp^vsi^-mRFP* (red).

The 14 TK24 derivative strains with deleted protease and protease inhibitor genes, were next characterized for the effect of protease gene deletions on growth and protein secretion. For this, the deletion mutant library was phenotyped toward growth-related parameters using a microbioreactor-based pipeline (Koepff et al., 2017). Most strains did not exhibit very strong phenotypes concerning the evaluated criteria (**Figure 2A**). Three of the four parameters μ_max_, CDW and cultivation time demand until stationary phase (t_batch_) directly quantified growth performance. These values vary in the deletion strains mostly within ∼20% to those of the WT. No deletion derivative strain showed consistently higher growth performance than that of TK24.

TK24Δ*ftsH* exhibited by far the strongest phenotype in all three parameters (**Figure 2A**). This highly transcribed membrane-embedded protease FtsH is an essential, hexameric, membrane-anchored metalloprotease in *E. coli* with a wide substrate diversity (Bieniossek et al., 2006; Walker et al., 2007). It targets multiple cellular processes including lipopolysaccharide biosynthesis, heat-shock sigma factor degradation, protein secretion, periplasmic chaperone functions and stress adaptation (Bittner et al., 2017). In comparison to TK24, TK24Δ*ftsH* revealed a 45% reduced μ_max_, coupled with 37% reduced CDW and a 53% prolonged t_batch_. FtsH3, a homologue of FtsH present in TK24 but absent from *E. coli*, was also deleted. However, this gene deletion has significantly less pronounced effects on growth than those of Δ*ftsH*. FtsH3 may have auxiliary roles in the cell that are less critical than those of FtsH.

### Secretome Analysis of Strains With Protease Deletions

**FIGURE 3:**
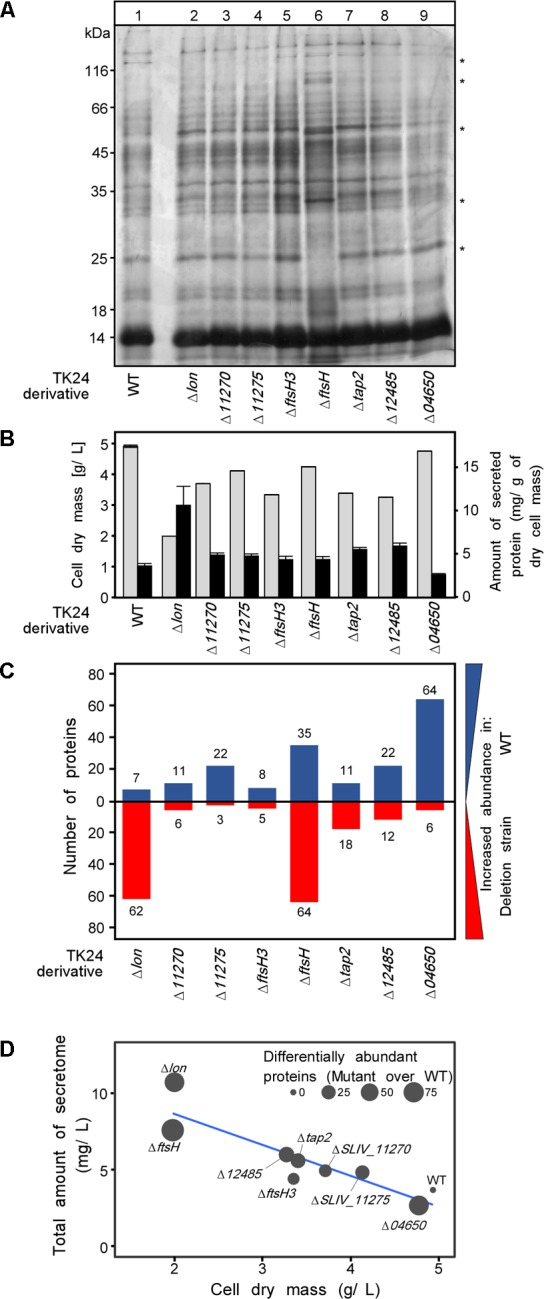
Secretome analysis of protease deletion strains. **(A)** Secretome profile of WT TK24 and derivatives resolved by SDS-PAGE and silver stained, loading equal amount of secretome polypeptides (3 μg). Representative samples from each TK24 derivative was loaded, for the strains that were used in the proteomic analysis. **(B)** Cell dry weight (g/L) (light gray) and amount of total proteins secreted (mg/L) (black) for the strains used in the proteomic analysis. **(C)** Number of differentially abundant secreted proteins between each TK24 derivative against the WT. Proteins with increased abundance in WT are shown in blue and proteins more abundant in deletion strains are colored red. Samples were loaded for proteomic analysis normalized to the same amount of cell biomass. **(D)** Correlation between dry cell weight, amount of total proteins secreted and number of differentially abundant proteins over the WT. (*n* = 4–7).

To test specifically the effect of the protease gene deletions on protein secretion we examined the secretomes of the derivative strains. Equal amounts of total secretome polypeptides were first analyzed by SDS-PAGE and silver staining (**Figure 3A**). The patterns of the various derivatives seemed similar overall at this level of analysis except for TK24Δ*ftsH* that gave rise to several different polypeptides and had lost others (**Figure 3A**, stars). Moreover, upon loading of secretome material derived from the same volume of culture, TK24Δ*ftsH* was also found to be a profuse secretor of polypeptides (not shown). As seen in other studies (Hamed et al., 2017; Tsolis et al., 2018), there appears to be a good correlation between suppressed growth and improved secretion as seen by comparison of the total secretome expressed per unit cell biomass (**Figure 3B**).

The same samples were also analyzed by label-free nanoLC-MSMS and the identity of the polypeptides determined and their amounts quantified (Supplementary Table S3). The abundance of proteins in the secretome of the deletion derivative strains was compared to that of the wild type (**Figure 3C**). In all cases, several polypeptides, representing 10–20% of the secretome, were identified at different abundances suggesting that the secretome is very sensitive to removal of proteases and yet, given the minor effect on fitness (**Figure 2A**), the cellular system remains robust (Supplementary Table S4). Functional characterization of these differentially abundant proteins, suggests that multiple hydrolases, proteins of housekeeping function and transport related proteins are oversecreted in the mutants showing the most severe phenotype (TK24Δ*ftsH*, TK24Δ*lon*) (Supplementary Figure S2 and Supplementary Table S3).

### Fitness- and Production Testing of Protease Deletion Strains Secreting a Heterologous Protein

In view of the wide-ranging effects of the deletion of protease genes on the secretome, we sought to corroborate these results with a more targeted approach that would also allow us to evaluate the potential of these strains for heterologous protein secretion. For this, protease deletion strains of interest were selected for a study focusing on the secretion of a single model protein SP^vsi^-mRFP (Hamed et al., unpublished). For this, the gene encoding SP^vsi^-mRFP that was previously stably integrated into the genome of TK24 (Hamed et al., unpublished) was now integrated in the same position of the genomes of all the individual TK24 derivative strains carrying the different protease and protease inhibitor gene deletions using the phage VWB attachment site as described (Hamed et al., unpublished).

*Streptomyces* species secrete various secondary metabolites, some of which fluoresce (Tenconi et al., 2013). It was therefore important prior to the SP^vsi^-mRFP secretion analysis to ensure that the background fluorescence of the various deletion strains was compatible with experimental detection and quantification of secreted SP^vsi^-mRFP. To this end a fourth parameter in the fitness/growth testing was the background fluorescence (fluor_background_) (**Figure 2A**). This was evaluated using an excitation/emission filter set (ex. 550 nm/em. 589 nm) appropriate for mRFP fluorescence detection and quantification. This analysis can identify possible fluorescence superposition effects, provoked by the various gene deletions that induced the production of endogenous fluorescent compounds by *Streptomyces* cells. However, none of the strains tested showed strongly enhanced background fluorescence with properties that could optically interfere with the fluorescence of SP^vsi^-mRFP. Therefore, TK24 derivatives with specific protease gene deletions were used to determine their effect on SP^vsi^-mRFP secretion.

The 14 strains with the integrated gene encoding SP^vsi^-mRFP were characterized, using the microbioreactor (**Figure 2B** and **Table 1**). All strains showed significantly increased biomass-specific mRFP fluorescence (fluor_CDWspecific_) in comparison to TK24. However, this parameter showed large variation (∼6 to ∼29-fold). TK24Δ*ftsH*::*SP^vsi^-mRFP* showed the highest biomass-specific mRFP fluorescence (∼29-fold) compared with TK24 (Supplementary Table S5). TK24::*SP^vsi^-mRFP* showed a ∼17-fold increase in comparison to the other strains that carried not only the SP^vsi^-mRFP reporter but also protease and protease inhibitor gene deletions.

To directly determine the extent to which the measured fluorescence represented secreted mRFP, we separated cells from spent growth media by centrifugation and measured the individual fluorescence in the two fractions. These data revealed that >89% of the mRFP fluorescence derived from secreted protein, in agreement with previous observations (Hamed et al., unpublished).

Additionally, we have tested the effect of deletion of the aforementioned genes on diamide tolerance in the presence and absence of the *SP^vsi^-mRFP* construct. Diamide is a thiol oxidant, causing generation of nonnative disulfide bonds, resulting in damage of cytoplasmic proteins (Hochgrafe et al., 2005). Most of the strains showed no or insignificant changes in diamide sensitivity. In contrast, the Δ*ftsH* and Δ*ftsH3* mutants were found to be more susceptible to the thiol oxidative stress when expressing the *SP^vsi^-mRFP* than the parental strain TK24 (Supplementary Figure S3 and Supplementary Table S6). At the same time, a small increase in sensitivity was observed also in the case of *S. lividans* deficient in SLIV_09985 encoding the putative integral membrane peptidase S8. This finding might indicate the involvement of these genes in protein quality control or protein processing, at least during growth on solid media.

### In-Depth Multi-Omics Analysis of the Strain Deleted for *ftsH*

Given the higher biomass-specific mRFP fluorescence performance of TK24Δ*ftsH*::*SP^vsi^-mRFP* and the significant effects on the secretome overall seen with TK24Δ*ftsH*, we further characterized the effects, caused by the deletion of this highly expressed, non-secreted core-protease.

**FIGURE 4:**
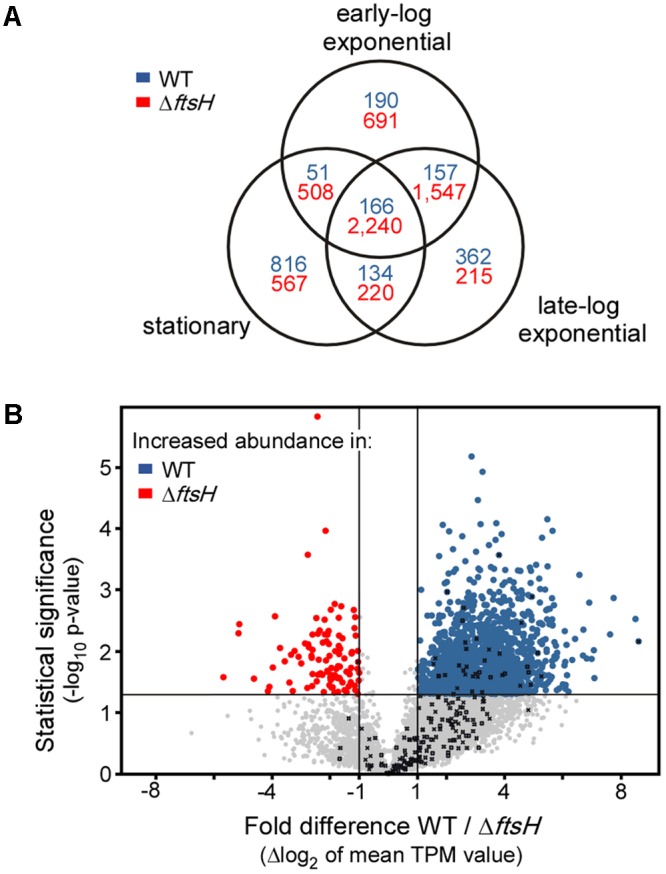
Transcriptomic activity of TK24Δ*ftsH* against that of TK24. **(A)** Venn diagram showing the number of genes with a change of transcript levels of at least twofold in the three growth stages sampled. Numbers in blue refer to genes with increased transcript abundance in the strain TK24Δ*ftsH* while numbers in red refer to genes with increased transcript abundance in the WT TK24. **(B)** Volcano-plot showing the differences in transcript abundance of all genes between strain TK24Δ*ftsH* and WT TK24. Each dot represents one gene. On the *x* axis is plotted the fold difference (in log_2_ scale) of the transcript abundance in the Δ*ftsH* strain compared to that in the WT, averaged over the three time points sampled. On the *y* axis, the *p*-value derived from a *t*-test between the two strains (–log_10_) is given. Transcripts with higher abundance in TK24Δ*ftsH* are given in blue, those more abundant in the WT are given in red, while the abundance of transcripts given in grey not significantly changed (–1 < m < 1 and or *p* > 0.05). Genes encoding proteases are listed in **Table 1**.

TK24Δ*ftsH* and TK24 were cultivated in the microbioreactor, to generate sufficient biological replicates for subsequent omics analysis. Samples were taken, during early and late growth phase, as well as during stationary phase, membrane filtered and snap-frozen. Given the role of *ftsH* in *E. coli* in regulating the turn-over of sigma-factors (Bittner et al., 2017) and the severity of the changes seen at the secretome level when it is deleted (**Figure 3**), it was of interest to determine whether some effect of Δ*ftsH* is already exerted at the transcriptome level. To test this, we compared the transcriptome profiles of TK24 with those of TK24Δ*ftsH* (**Figure 4**). This analysis revealed that loss of FtsH has a significant effect on the transcriptome, regardless of the growth phase sampled. Overall, the transcript abundance of 2,240 genes was reduced at least twofold in all three growth phases sampled, while only 166 genes showed a consistent increase in transcript abundance (**Figure 4A**). This reduction in transcript levels was even more pronounced in the early and late-log phase (an additional 1,547 genes with reduced pools compared to an additional 157 genes with increased pools), while the stationary phase alone shows a significant increase in some transcript pools (816 genes). This major disturbance of the transcriptome seems to correlate with the reduced growth of TK24Δ*ftsH*, but makes it nearly impossible to pinpoint any specific molecular cause. This becomes apparent when the average change in transcript levels over all three time points analyzed is examined (**Figure 4B**): With more than 1,567 genes with significantly changed transcript pools (1,466 with decreased pools and 101 with increased pools), the effects of the *ftsH* deletion can only be described as global.

**FIGURE 5:**
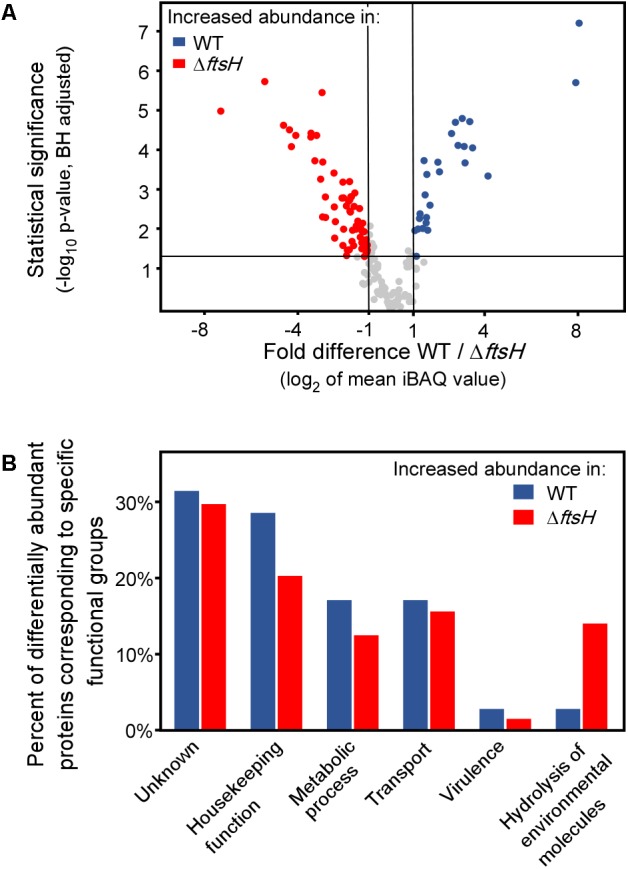
Comparative secretomics analysis of TK24Δ*ftsH* against TK24. **(A)** Volcano-plot showing the differentially abundant proteins between TK24 and TK24Δ*ftsH*. Each dot represents one protein. On the *x* axis is plotted the fold difference (in log_2_ scale) of the mean protein abundance in the TK24 (marked: WT) over that in TK24Δ*ftsH*, and on the *y* axis the *p*-value derived from a *t*-test between the two strains (–log_10_, adjusted by the Benjamini–Hochberg method). With blue are colored proteins more abundant in the WT and in red, the significantly more abundant proteins in TK24Δ*ftsH.* **(B)** Functional classification of differentially abundant proteins based on their biological function. The ratio of the abundant proteins corresponding to a specific functional category over the number of over-represented proteins in the specific strain is plotted.

Finally, we analyzed the secretome of TK24Δ*ftsH* and compared it to that of the wild type. The abundance of 99 secreted proteins is statistically different in the two strains, reflecting proteins that are seen at both lower and higher levels in the mutant strain (**Figure 5A**). Proteins affected include: a quinoprotein amine dehydrogenase, a solute-binding lipoprotein and a D-alanyl-D-alanine carboxypeptidase that are synthesized/secreted 3-7 times less than in TK24 and a Phospholipase-A2 domain-containing protein, a spore-associated protein A and a branched chain amino acid binding protein that are secreted > 4 times more. Overall, the affected proteins fall in 6 main functional classes (**Figure 5B**). This is suggestive of an extensive regulatory role of *ftsH* in *S. lividans.*

## Discussion

Our analysis aimed to determine whether proteases play a role in protein secretion and whether disruption of the secreted protease equilibria affects the secretome of *S. lividans* overall. Our data suggested that deletion of multiple proteases is possible in *S. lividans*. While, significant qualitative and quantitative effects can be seen on the secretome, in most cases the strains maintain metabolic robustness.

The effect of the various deletions on the secretome would indicate that secretome proteases are under strict metabolic control, although the molecular mechanism underlying this regulation remains unknown. Nitrogen provided by the casamino acids may down-regulate the synthesis of some of the proteases until this resource is depleted. The apparent clustering pattern of several of the proteases (**Figure 1B**) also raises the possibility that they may be under the same transcriptional regulatory control. The multiplicity of sigma and anti-sigma factors in TK24 (Luzhetskyy et al., unpublished) and the currently incomplete understanding of promoter usage and promiscuity, renders buildup of regulon networks challenging. Another parameter that would regulate these equilibria would be protease inhibitors, particularly given that many of them will inhibit multiple proteases (e.g., there are 6 secreted subtilisin-like proteases that may be inhibited by the same molecules). Better understanding of these networks is expected to derive from RNAseq analyses combined with strains deleted for specific sigma factors.

Despite the obvious qualitative and quantitative changes observed, overall most of the deletions had no obvious effect on fitness under the conditions tested. This is suggestive of a high degree of build-in redundancy and/or the peripheral contribution of the removed secreted proteases in house-keeping processes of the cellular network. This was also true for removal of the cytoplasmic and membrane proteases of known regulatory importance in cell proteostasis such as Lon and FtsH3 (**Figure 3**).

Some secretory proteases would be expected to have regulatory roles. For example, serine proteases were reported to coordinately regulate the cellular protein turnover associated with secondary metabolism and morphogenesis (Taguchi et al., 1995), also, their physiological roles in terms of mycelial growth, autolysis of mycelia after stationary phase in submerged cultures (Kim and Lee, 1995). Furthermore, leupeptin-inactivating enzyme also seems to play a critical role in mycelium differentiation of *S. exfoliates* SMF13 by controlling the amount of leupeptin that regulates trypsin-like proteases activity (Kim et al., 1998). In these cases, their removal would lead to the stable accumulation of their potential polypeptide-targets that now become more stable and can be detected. In some cases, secreted polypeptides of enhanced abundance were indeed observed. However, in addition, certain secreted polypeptides also became less abundant when specific proteases were removed (**Figure 4B** and Supplementary Table S3). This would suggest that some secreted proteases are co-regulated together with other secreted polypeptides in a way that is independent of the proteolytic activity. The molecular basis of these apparently complex balanced networks remains unknown.

FtsH, was the only protease with a significant effect on fitness (**Figure 3**, lane 6). While, the biological function of FtsH and the need for a second copy in *Streptomyces* is not well understood, we assume that the severity of the effect, reflects important regulatory roles in TK24 as seen previously in *E. coli* (Langklotz et al., 2012). This is further corroborated by the major disturbance of the transcriptome and secretome of TK24Δ*ftsH*, and hence, FtsH is expected to be a major global regulator affecting 6 main functional classes of secretome proteins (**Figure 5B**). The regulatory role of *ftsH* in *S. lividans* may be exerted through a combination of mechanisms that involve both transcriptional (**Figure 4**) and other means of regulation, e.g., protein degradation. Little is known about the molecular basis of these effects and how they might be inter-connected. The extent of the transcriptome alterations in TK24Δ*ftsH* precludes any guidance as to a specific molecular pathway. At least two hypotheses can be entertained: (a) as FtsH is membrane-embedded and secretion is a process of transmembrane-crossing, the actual translocation of some proteins or their clearing from translocase sites may require FtsH, as seen in *E. coli* (Langklotz et al., 2012). (b) removal of FtsH may bring the cell to a stress state that could be analogous to the role of FtsH in dealing with membrane protein stress in *E. coli* (Bittner et al., 2017). This may correlate with transcripts of known stress-related genes such as SLIV_24510 (encoding superoxide dismutase), SLIV_12445 (encoding a glutaredoxin-like protein), being 2-45-fold elevated in TK24Δ*ftsH*. Whichever the mechanism, FtsH appears to be a core proteostatic component since no other proteases can replace it and maintain the same transcript level trends as those of the other genes (**Figure 4B**, marked with black Xs).

The stable activity of secreted mRFP underlines the suitability of *S. lividans* for heterologous protein production and is reminiscent of what has been observed with several other heterologous proteins (Pozidis et al., 2001; Sianidis et al., 2006; Hamed et al., 2017). Perhaps the deletion of multiple secreted proteases could be more beneficial for heterologous protein production, but this can be seen now under new light. Previously, multiple deletions in Gram-positive bacteria were used to reduce proteolysis of the heterologous product [e.g., in *B. subtilis* (Westers et al., 2004)]. Furthermore, deletion of 8 secreted proteases in *B. subtilis* affects the heterologous production not only by reducing its degradation but by increasing the extra-cytoplasmic chaperons and quality control factors PrsA, HtrA and HtrB as well (Krishnappa et al., 2013, 2014). However, we now see that removal of proteases can also act in a regulatory role. This is more prominently seen with deletion of genes for the regulatory protease FtsH, which has a role in quality control of membrane proteins (Dalbey et al., 2012). Therefore, a finer regulation of proteases such as FtsH might have implication for improving the quality and secretion yield.

## Materials and Methods

### Generation of Protease Gene Deletions

To delete selected genes BAC clones from ordered *S. lividans* BAC library were selected and mutagenized using the Red/ET technique combined with the apramycin resistance IMES cassette from patt-saac-oriT (Myronovskyi et al., 2014). Primers used to amplify the cassette and to verify the mutation are listed in Supplementary Table S7. Red/ET recombineering of the BACs using amplified apramycin resistance IMES cassette fragment was performed as described previously (Gust et al., 2004). The resulting recombinant BACs were introduced in the *S. lividans* TK24 via conjugation (Rebets et al., 2017). Screening for double-crossover mutants was performed on mannitol soya flour (MS) medium (per liter: 20 g agar, 20 g mannitol, 20 g soya flour) supplemented with 50 μg/ml of apramycin and 70 μg/ml of X-gluc. Gene deletions were confirmed via PCR using appropriate check primers.

The fragment containing the *SP^vsi^-mRFP* gene, i.e., the *S. venezuelae* subtilisin inhibitor signal peptide *SP^vsi^* fused to *mRFP* behind the strong *vsi* promotor (*P_vsi_*) was excised from the plasmid pIJ486-SP^vsi^-mRFP (Hamed et al., unpublished) using *Xba*I and *Hind*III and ligated into the respective sites of pTOS (Herrmann et al., 2012) yielding pTOS + mRFP that contained the SP^vsi^-mRFP-encoding gene and *attB* of VWB-phage flanked by two *rox*-sites. This plasmid was introduced into the genome of *S. lividans* TK24 and protease gene deletion strains by intergeneric conjugation with *E. coli* ET1326::pUZ8002 (Kieser et al., 2000) as described (Herrmann et al., 2012). For each conjugated strain, genomic DNAs of four randomly chosen exconjugants were isolated and verified by PCR for proper integration of the pTOS + mRFP plasmid. Then, a plasmid containing the gene of the *Dre* recombinase (pUWLDre), was introduced into the respective mutant strains and the pTOS + mRFP-backbone was excised as described (Herrmann et al., 2012).

### Rapid *S. lividans* Strain Phenotyping

Time-efficient characterization of the strain library was realized using a previously published workflow, which utilizes parallelized microbioreactor cultivation in 48-well microplate with a working volume of 1000 μL in each well (Koepff et al., 2017). All strains were, at minimum, cultivated in biological triplicates. The WT was incorporated in each separate microtiter-plate run. The corresponding WT results were averaged and used to normalize the data, obtained from all other deletion mutants. By this methodology, batch-related differences could be compensated. Detailed cultivation and data processing pipeline is described in detail in (Luzhetskyy et al., unpublished).

Monomeric red fluorescence protein fluorescence was relatively quantified during cultivation by using a excitation/emission: 550 nm/589 nm filter set, incorporated in the automated microbioreactor (m2p-labs, Baesweiler, Germany). To efficiently compare the strain performance, mRFP fluorescence intensity values, obtained at the transition to stationary phases were identified and evaluated. Error propagation was applied to calculate CDW-specific mRFP production.

### Lab-Scale Bioreactor Cultivation

Lab-scale cultivations of *S. lividans* were carried out in parallelized glass bioreactors (DasGip, Jülich, Germany) with a working volume of 1000 mL. In principle, the same media composition as in the microbioreactor was applied, with the exception, that no MES buffer was used, but a constant pH of 6.8 was maintained by titrating 4 M NaOH or 4 M HCl solutions if required.

### Transcriptomics, Identification of Highly Transcribed Proteases

Samples were taken during the early and late log growth phase as well as after entry into the stationary phase. Harvesting and RNA isolation was performed as described previously (Busche et al., 2012). Samples from different biological replicates were isolated separately and pooled after quality control. The RNA quality was checked via Agilent 2100 Bioanalyzer (Agilent Technologies, Böblingen, Germany) and Trinean Xposesystem (Gent, Belgium) prior and after rRNA depletion using the Ribo Zero rRNA Removal Kit for Bacteria (Epicentre, Madison, WI, United States). The TruSeq Stranded mRNA Library Prep Kit from Illumina was used to prepare the cDNA libraries, which were then sequenced in paired-end mode on an Illumina HiSeq 1500 system with 28 respectively 70 bases read length.

Transcripts per kilobase million (TPM) (Wagner et al., 2012) were calculated using READXPLORER v.2.2 (Hilker et al., 2016). For differential RNA-Seq analyses the signal intensity value (*A*-value) was calculated by average log_2_(TPM) of each gene and the signal intensity ratio (*M*-value) by the difference of log_2_ (TPM). In cases where the TPM for a gene was 0, a TPM of 0.1 was used instead to avoid log_2_(0). To identify proteases that were strongly transcribed and differentially expressed under at least one condition, the RNA-Seq data were filtered using a TPM cut-off of 100 and an M-value cut-off of >1.0 under at least one condition. The raw sequence data sets are available at the NCBI SRA under study ID SRP144344, SRA accessions SRR7093716-SRR7093727.

For analysis of the transcriptome comparisons of the Δ*ftsH* and WT strains, the average of the *M*-values over all three time points sampled as well as a *P*-value based on the log_2_(TPM) values using a Student’s *t*-test (two tailed, heteroscedastic) were calculated. Genes with an average *M*-value above/below 1/-1 and a *P*-value < 0.05 were considered to be differentially transcribed.

### Secretomics Sample Preparation and Measurement

Cells were removed by centrifugation (4,500 × *g*; 5 min; 4°C) and subsequent filtration (syringe filter, 0.2 μm, cellulose acetate). Proteins contained in culture supernatants were precipitated via 25% v/v TCA precipitation (4°C; 20 min). Precipitated proteins were pelleted via centrifugation (20,000 × *g*; 20 min; 4°C), on a bench-top centrifuge. The pellet was washed twice with ice-cold acetone and re-pelleted via centrifugation (20,000 × *g*; 20 min; 4°C). The protein pellet was then solubilized in 8 M Urea in 1 M ammonium bicarbonate solution (ABS). Polypeptide concentrations were measured using the Bradford reagent. Polypeptides (3 μg) were separated by 12% SDS-PAGE and visualized by silver staining (Shevchenko et al., 1996).

### Analysis of Secretomes by NanoLC-MS/MS

A volume corresponding to the secreted polypeptides derived from 3 × 10^6^ cells (usually a volume equivalent to 20–40 μL of the initial cell culture) was used for in-solution tryptic digestion. The protein solution was initially diluted into urea (2 M final concentration in 50 mM ABS, followed by reduction of cysteines with 1 mM DTT (45 min; 56°C), alkylation using 10 mM Iodoacetamide (IAA) (45 min; 22°C; dark) and digestion using 0.015 μg Trypsin for 1.5 μg protein (Trypsin Gold, Promega, Fitchburg, WI, United States; ratio trypsin/protein 1/100; overnight; 37°C). Digested peptide solutions, were acidified with trifluoroacetic acid (TFA) to pH < 2, desalted using STAGE tips (Rappsilber et al., 2007; Tsolis and Economou, 2017), and stored lyophilized at -20°C, until the MS analysis.

Lyophilized peptide samples were re-suspended in an aqueous solution containing 0.1% v/v formic acid (FA) and 5% v/v ACN and analyzed using nano-Reverse Phase LC coupled to a QExactive Hybrid Quadrupole – Orbitrap mass spectrometer (Thermo Scientific, Bremen, Germany) through a nanoelectrospray ion source (Thermo Scientific, Bremen, Germany). Peptides were initially separated using a Dionex UltiMate 3000 UHPLC system on an EasySpray C18 column (Thermo Scientific, OD 360 μm, ID 50 μm, 15 cm length, C18 resin, 2 μm bead size) at a flow rate of 300 nL min^-1^. The LC mobile phase consisted of two different buffer solutions, an aqueous solution containing 0.1% v/v FA (Buffer A) and an aqueous solution containing 0.08% v/v FA and 80% v/v ACN (Buffer B). A 60 min multi-step gradient was used from Buffer A to Buffer B as follows [0–3 min constant (96:4), 3–15 min (90:10); 15–35 min (65:35); 35–40 min (35:65); 40–41 min (5:95); 41–50 min (5:95); 50–51 min (95:5); 51–60 min (95:5)].

The separated peptides were analyzed in the Orbitrap QE operated in positive ion mode (nanospray voltage 1.5 kV, source temperature 250°C). The instrument was operated in DDA mode with a survey MS scan at a resolution of 70,000 FWHM for the mass range of m/z 400–1600 for precursor ions, followed by MS/MS scans of the top 10 most intense peaks with +2, +3, and +4 charged ions above a threshold ion count of 16,000 at 35,000 resolution. MS/MS was performed using normalized collision energy of 25% with an isolation window of 3.0 m/z, an apex trigger 5–15 s and a dynamic exclusion of 10 s. Data were acquired with Xcalibur 2.2 software (Thermo Scientific).

Raw MS files were analyzed by the MaxQuant v1.5.3.3 proteomics software package (Cox and Mann, 2008). MS/MS spectra were searched by the Andromeda search engine against the Uniprot *S. lividans* TK24 proteome (taxonomy: 457428, last modified May, 2016, 7320 protein entries; (Ruckert et al., 2015) and common contaminants (e.g., trypsin, keratins). Enzyme specificity was set to trypsin and a maximum of two missed cleavages were allowed. Dynamic (methionine oxidation and N-terminal acetylation) and fixed (*S*-carbamidomethylation of cysteinyl residues) modifications were selected. Precursor and MS/MS mass tolerance was set to 20 ppm for the first search (for the identification of the maximum number of peptides for mass and retention time calibration) and 4.5 ppm for the main search (for the refinement of the identifications). Protein and peptide FDR were set to 1%. FDR was calculated based on the number of spectra matched to peptides of a random proteome database (reversed sequence database) in relation to the number of spectra matching to the reference proteome. Peptide features were aligned between different runs and masses were matched (“match between runs” feature), with a match time window of 3 min and a mass alignment window of 20 min. Protein quantification was performed using the iBAQ algorithm (Schwanhausser et al., 2011) through MaxQuant software. Differentially abundant proteins were selected using the *t*-test and by comparing the fold difference of average protein intensities between the samples. *P*-values were further corrected for multiple hypothesis testing error using the BH method (Benjamini and Hochberg, 1995). Thresholds for the analysis were set to adjusted *p*-value < 0.05 and fold difference > 2. Functional characterization of proteomics results was performed after filtering the dataset only to secreted proteins, excluding cytoplasmic contamination, using proteome annotation as described in the SToPSdb (Tsolis et al., 2018)^2^. The percentage of differentially abundant proteins that match a specific term over the total differentially abundant proteins for each condition was plotted. Keywords were derived after manual curation of the proteome.

### Miscellaneous

Chemicals were from Sigma. DNA enzymes were from New England Biolabs and oligonucleotides from Eurogentec. The mass spectrometry proteomics data have been deposited to the ProteomeXchange Consortium via the PRIDE partner repository with the dataset identifiers PXD006819 (Vizcaino et al., 2016).

## Author Contributions

YR and AL constructed the protease gene deletion strains. ML performed deletion of protease-encoding genes. AY deleted the markers from protease mutants and introduced the *SP^vsi^-mRFP* construct. JK, WW, and MO performed microbioreactor growth and mRFP measurements for mutated strains and analyzed data. TB, CR, and JK performed the transcriptomics experiments and analyzed the data. MH and KT performed proteomics experiments and analyzed the data. JA and SK analyzed the data. AE managed and supervised the project and wrote the paper with contributions from SK, JA, MH, KT, CR, AL, and TB. All authors read and approved the manuscript.

## Conflict of Interest Statement

The authors declare that the research was conducted in the absence of any commercial or financial relationships that could be construed as a potential conflict of interest.

## Footnotes

1 www.stopsdb.eu

2 www.stopsdb.eu

## Abbreviations

μ_max_: maximum specific growth rate
ACN: Acetonitrile
BH: Benjamini-Hochberg
CDW: cell dry weight concentration
DDA: Data-dependent acquisition
em.: emission wavelength [nm]
ex.: excitation wavelength [nm]
FDR: false discovery rate
fluor_background_: background mRFP fluorescence
fluor_CDWspecific_: cell-dry-weight concentration specific mRFP fluorescence
FWHM: Full-width half maximum
mRFP: monomeric red fluorescence protein
t_batch_: batch time: cultivation time demand, required to reach stationary phase
TFA: trifluoroacetic acid
WT: *S. lividans* TK24 wild-type
