# Supplementary material for: Multi-Omics and Targeted Approaches to Determine the Role of Cellular Proteases in Streptomyces Protein Secretion
Source: Front Microbiol. 2018 Jun 4;9:1174. doi: 10.3389/fmicb.2018.01174 (PMC5994538; doi:10.3389/fmicb.2018.01174)
Supplement: Supplementary file 4 [file Data_Sheet_1.PDF]

Supplementary Information

Multi-Omics and Targeted Approaches to Determine the Role of Cellular Proteases in *Streptomyces* Protein Secretion

Busche, Tobias[1,6*], Tsolis, C. Konstantinos[2*], Koepff, Joachim[3], Rebets, Yuriy[4,5], Rückert, Christian[1], Hamed, B. Mohamed[2,7], Bleidt, Arne[3], Wiechert, Wolfgang[3], Lopatniuk, Mariia[5], Ahmed, Yousra[5], Jozef Anné[2], Karamanou, Spyridoula[2], Oldiges, Marco[3,8], Kalinowski, Jörn [1], Luzhetskyy, Andriy[4] and Economou, Anastassios[2,9]

[1]Center for Biotechnology (CeBiTec), Bielefeld University, Bielefeld, Germany.

[2]KU Leuven, Rega Institute, Dpt of Microbiology and Immunology, Herestraat 49, B-3000 Leuven, Belgium.

[3]Forschungszentrum Jülich GmbH, Institute of Bio- and Geosciences, IBG-1: Biotechnology, Leo-Brandt-Straße, 52428, Jülich, Germany.

[4]Helmholtz-Zentrum für Infektionsforschung GmbH, Inhoffenstraße 7, 38124 Braunschweig, Germany.

[5]Universität des Saarlandes, Pharmazeutische Biotechnologie, Campus C2.3, Saarbrücken, 66123 Germany

[6]Institute for Biology-Microbiology, Freie Universität Berlin , Berlin, Germany

[7]Molecular Biology Dpt, National Research Centre, Dokki, Giza, Egypt.

[8]RWTH Aachen University, Institute of Biotechnology, Worringer Weg, Aachen, Germany

* Equal contribution

[9]For correspondence:

e-mail: tassos.economou@kuleuven.be

Table of content:

1- Supplementary Figures

Figure S1. Correlation of relative secreted protease abundance between transcriptomics and proteomics. Proteases were ranked from the most abundant (rank 1) to the least abundant based on their mean transcript (TPM values) and protein (iBAQ values) quantification of the WT *S. lividans* TK24 in MM-CAS.

Figure S2. Diamide sensitivity of *S. lividans* strains in presence of sp^{vsi}*-mRFP* construct. Upper panel – MS medium, lower panel – TSB agar medium.

2- Supplementary Tables

Table S1. List of proteases encoded in *S.lividans* TK24

Table S2. Proteins identified by MS.

Table S3. Differentially synthesized proteins.

Table S4. Strain phenotyping data of protease deletion strain versions before mRFP integration.

Table S5. Strain phenotyping data of protease deletion strain versions after mRFP integration.

Table S6. Diamide sensitivity of *S. lividans* TK24 and generated mutants expressing sp^{vsi}-*mRFP*.

Table S7. Primers used in this work.

3-Supplementary Methods

- **Analysis of diamide sensitivity.**

4- References

1- Supplementary Figure:

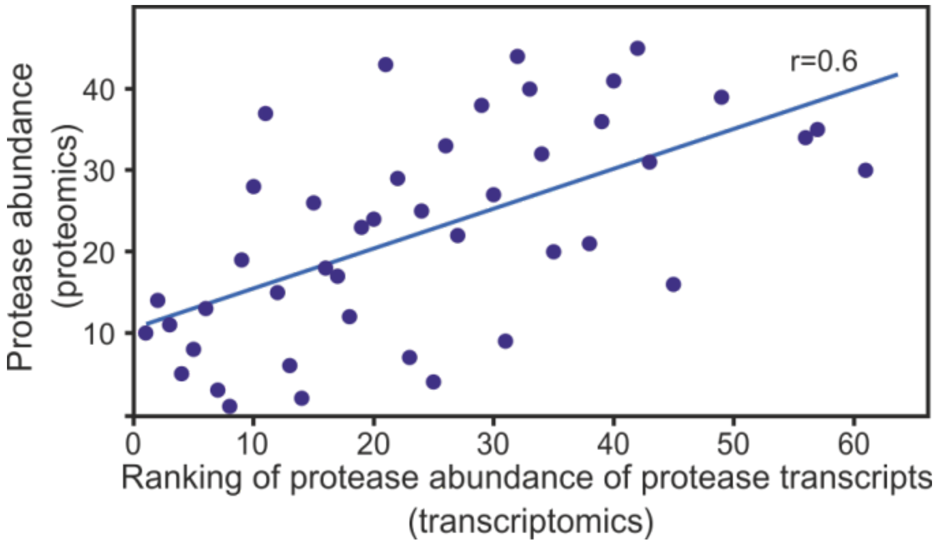

Figure S1. Correlation of relative secreted protease abundance between transcriptomics and proteomics.

Proteases were ranked from the most abundant (rank 1) to the least abundant based on their mean transcript (TPM values) and protein (iBAQ values) quantification of the WT *S. lividans* TK24 in MM-CAS.

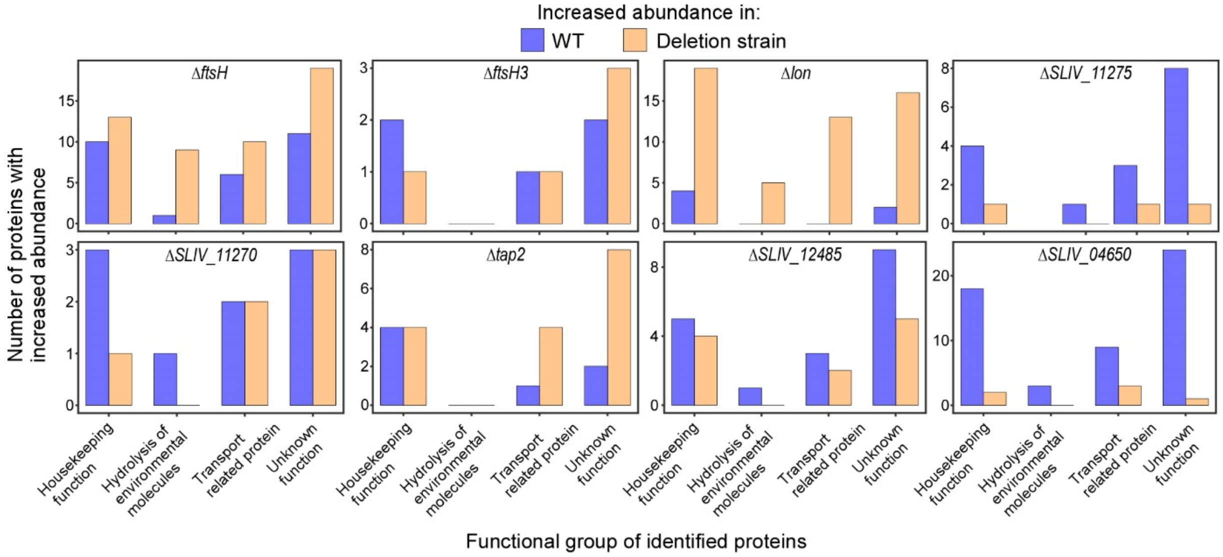

Figure S2. Functional characterization of protease deletion secretome.
Differentially abundant proteins between the WT and the protease deletion strain are divided into groups based on their function (Tsolis et al., 2018). Dataset is filtered to secreted proteins. Cytoplasmic contaminating proteins are removed.

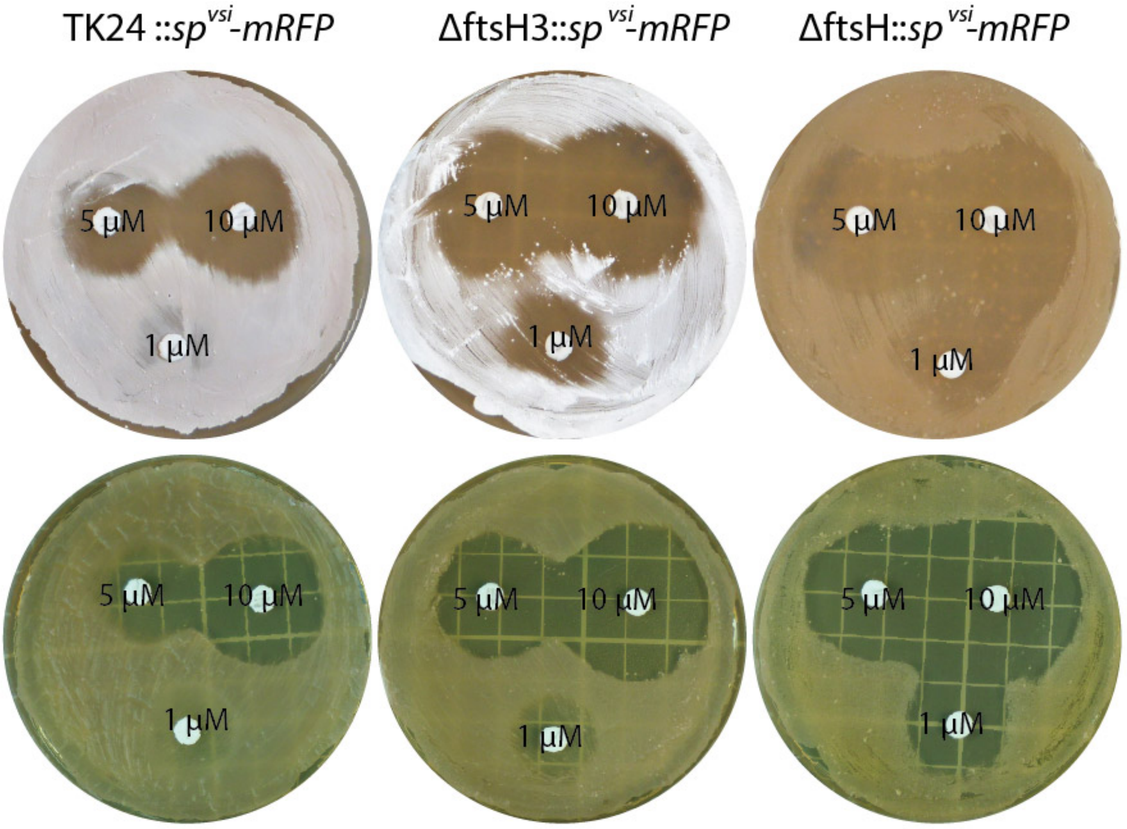

Figure S3. Diamide sensitivity of *S. lividans* strains in presence of *sp*^{vsi}-*mRFP* construct.
Upper panel – MS medium, lower panel – TSB agar medium.

2- Supplementary Tables:

Table S1: List of proteases encoded in *S.lividans* TK24,

This list is based on the latest genome and proteome annotation which contains proteases detected by transcriptomics and proteomics and their relative ranking based on abundance.
Table S1 is provided in the additional spreadsheet file:
"Table S1 S. lividans TK24 proteases_v03.xlsx".

Table S2: Proteins identified by MS.

Proteins identified by mass spectrometric workflow (see Methods). Quantitative values (determined using iBAQ), Sequence coverage for the identified proteins and number of peptides per protein for each biological sample are included.
Table S2 is provided in the additional spreadsheet file:
"Table S2_Proteins identifed by mass spectrometry".

Table S3: Differentially synthesized proteins.

Comparison of WT vs deletion strain for differentially synthesized proteins, for the secreted proteins based on annotation in the SToPS database http://www.stopsdb.eu (Tsolis et al., 2018).
Table S3 is provided in the additional spreadsheet file:
"Table S3_Differentially abundant secreted proteins between WT and protease deletion strain".

Table S4: Strain phenotyping data of protease deletion strain versions before mRFP integration. Maximum specific growth rate (μ_{max}), cell-dry-weight concentration at the transition to stationary phase (CDW) as well as cultivation time duration until this point (t_{batch}) and background fluorescence at excitation 550 nm and emission 589 nm ($fluor_{background}$) are provided as values and standard deviation for biological replicates of each strain and relative to the wild-type (WT) of the corresponding experiment. See the methods section for detailed methodology.

ID Jülich	Deleted gene locus	Deleted gene product	mRFP integration	μ_{max}		CDW		t_{batch}		$fluor_{background}$	
				value	range	value	std	value	std	value	std
SL000	-	-	-	1,00	0,03	1,00	0,07	1,00	0,07	1,00	0,09
SL318	SLIV_09985	Integral membrane Peptidase S8, subtilisin-related protein	-	0,86	0,02	0,96	0,00	1,06	0,06	0,77	0,00
SL317	SLIV_20750	Integral membrane ATP-dependent zinc metalloprotease FtsH (EC 3.4.24.-)	-	0,55	0,05	0,63	0,02	1,53	0,15	0,61	0,06
SL316	SLIV_10535	Integral membrane ATP-dependent zinc metalloprotease FtsH3	-	0,84	0,06	1,02	0,02	1,06	0,13	0,88	0,01
SL315	SLIV_11275	Secreted Neutral zinc metalloprotease	-	0,92	0,03	1,05	0,01	1,05	0,04	0,82	0,03
SL314	SLIV_11270	Secreted Neutral zinc metalloprotease	-	0,93	0,03	0,81	0,00	0,99	0,01	0,78	0,02
SL329	SLIV_15325	Secreted Peptidase, Leupeptin-inactivating enzyme 1	-	0,89	0,06	0,84	0,04	1,14	0,00	0,87	0,02
SL328	SLIV_09410	Secreted peptidase	-	0,84	0,05	1,03	0,02	1,23	0,03	1,00	0,02
SL327	SLIV_24720	Secreted Protein containing Tachylectin 2 domain	-	0,81	0,03	0,97	0,01	1,19	0,05	0,96	0,03
SL326	SLIV_34120	Secreted Probable subtilase-type protease inhibitor	-	0,75	0,01	0,87	0,07	1,40	0,04	1,14	0,02
SL320	SLIV_28740	Integral membrane Stomatin family	-	0,91	0,04	1,03	0,03	1,01	0,11	0,86	0,05
SL307	SLIV_02150	Secreted extracellular small neutral protease	-	0,97	0,01	0,75	0,04	0,96	0,02	0,90	0,06
SL321	SLIV_17030	Secreted Peptidase M1, alanine aminopeptidase/leukotriene A4 hydrolase	-	0,98	0,02	0,84	0,02	1,07	0,06	0,87	0,02
SL319	SLIV_10025	Integral membrane T7SS peptidase S8A, mycosin-1, component of T7S export system	-	0,88	0,04	0,84	0,05	1,11	0,04	0,83	0,01
SL309	SLIV_11935	Cytoplasmic ATP-dependent serine protease Lon	-	0,97	0,03	1,01	0,01	0,91	0,01	0,87	0,02

Table S5: Strain phenotyping data of protease deletion strain versions after mRFP integration. Annotation is identical to that in Table S1, except for mRFP fluorescence that is provided as CDW-specific values and standard deviation ($fluor_{CDWspecific}$). All values are normalized aginst those of the WT strain that does not harbor the integrated mRFP gene.

ID Jülich	Deleted gene locus	Deleted gene product	mRFP integration	μ_{max}		CDW		t_{batch}		$fluor_{CDWspecific}$	
				value	range	value	std	valu e	std	value	std
SL000	-	-	-	1,00	0,07	1,00	0,01	1,00	0,06	1,00	0,13
SL348	-	-	x	0,86	0,06	1,01	0,03	1,13	0,08	16,95	1,32
SL350	SLIV_09985	Integral membrane Peptidase S8, subtilisin-related protein	x	0,82	0,02	1,05	0,02	1,04	0,05	14,64	0,22
SL351	SLIV_20750	Integral membrane ATP-dependent zinc metalloprotease FtsH (EC 3.4.24.-)	x	0,64	0,06	0,43	0,02	1,54	0,02	29,53	0,44
SL352	SLIV_10535	Integral membrane ATP-dependent zinc metalloprotease FtsH3	x	0,84	0,05	0,92	0,01	1,11	0,07	19,31	1,43
SL353	SLIV_11275	Secreted Neutral zinc metalloprotease	x	0,96	0,04	1,16	0,03	1,03	0,02	11,42	0,44
SL354	SLIV_11270	Secreted Neutral zinc metalloprotease	x	0,82	0,03	1,10	0,01	0,99	0,02	11,20	0,32
SL355	SLIV_15325	Secreted Peptidase, Leupeptin-inactivating enzyme 1	x	0,94	0,05	1,13	0,01	0,95	0,03	7,92	0,93
SL356	SLIV_09410	secreted peptidase	x	0,77	0,07	0,95	0,05	1,12	0,09	15,75	0,54
SL357	SLIV_24720	secreted Protein containing Tachylectin 2 domain	x	0,83	0,02	1,07	0,01	1,00	0,02	13,28	0,23
SL358	SLIV_34120	Secreted Probable subtilase-type protease inhibitor	x	0,85	0,07	0,99	0,02	1,07	0,08	15,82	0,53
SL359	SLIV_28740	Integral membrane Stomatin family	x	1,05	0,07	0,79	0,03	1,02	0,05	15,81	3,87
SL360	SLIV_02150	Secreted extracellular small neutral protease	x	0,86	0,06	1,06	0,07	1,01	0,06	10,42	0,92
SL361	SLIV_17030	Secreted Peptidase M1, alanine aminopeptidase/leukotriene A4 hydrolase	x	0,92	0,05	0,91	0,00	0,98	0,03	13,66	2,37
SL362	SLIV_10025	Integral membrane T7SS peptidase S8A, mycosin-1, component of T7S export system	x	0,76	0,06	0,91	0,06	1,20	0,04	16,63	1,01
SL363	SLIV_11935	Cytoplasmic ATP-dependent serine protease Lon	x	0,96	0,01	1,20	0,00	0,97	0,04	6,07	0,33

Table S6: Diamide sensitivity of *S. lividans* TK24 and generated mutants expressing *sp*^{vsi}*-mRFP*. Growth inhibition (in mm) caused by diamide of *S. lividans* TK24 derivative mutant strains with or without the integrated *sp*^{vsi}*-mRFP* expression construct was determined by the disc diffusion assay. n=3; values represent mean±SD.

**ID Jülich**	**Deleted gene locus**	**Deleted gene product**	**TSB**				**MS**				Medium
			5 µM		10 µM		5 µM		10 µM		diamide
			-	+	-	+	-	+	-	+	*sp*^{vsi}-mRPF
SL000	-	-	21±3	22±3	26±4	29±3	15±2	17±2	22±1	24±1	
SL350	SLIV_09985	Integral membrane Peptidase S8, subtilisin-related protein	23±3	27±4	30±4	31±4	22±3	23±3	27±3	31±6	
SL351	SLIV_20750	Integral membrane ATP-dependent zinc metalloprotease FtsH (EC 3.4.24.-)	18±3	**31±5**	23±3	**38±4**	16±1	**30±3**	22±3	**37±3**	
SL352	SLIV_10535	Integral membrane ATP-dependent zinc metalloprotease FtsH3	**25±3**	**28±5**	**29±3**	**35±4**	**18±2**	**25±3**	**27±2**	**29±2**	
SL353	SLIV_11275	Secreted Neutral zinc metalloprotease	21±3	21±3	25±3	29±3	16±2	18±2	23±1	25±2	
SL354	SLIV_11270	Secreted Neutral zinc metalloprotease	19±2	22±3	24±5	30±3	15±2	18±2	22±1	25±2	
SL355	SLIV_15325	Secreted Peptidase, Leupeptin-inactivating enzyme 1	20±2	21±2	27±5	30±3	17±2	19±2	23±1	25±1	
SL356	SLIV_09410	secreted peptidase	21±2	21±2	27±4	29±3	16±1	18±2	25±2	26±1	
SL357	SLIV_24720	secreted Protein containing Tachylectin 2 domain	20±2	21±3	26±3	28±2	18±1	23±3	25±3	27±2	
SL358	SLIV_34120	Secreted Probable subtilase-type protease inhibitor	21±2	23±3	27±4	31±3	17±2	23±3	22±2	30±3	
SL359	SLIV_28740	Integral membrane Stomatin family	22±2	25±4	26±3	31±4	16±1	18±2	21±3	26±2	
SL360	SLIV_02150	Secreted extracellular small neutral protease	21±2	24±3	28±4	29±3	16±1	18±1	24±2	26±2	
SL361	SLIV_17030	Secreted Peptidase M1, alanine aminopeptidase/leukotriene A4 hydrolase	21±3	25±3	30±3	31±2	19±4	23±4	29±4	31±4	
SL362	SLIV_10025	Integral membrane T7SS peptidase S8A, mycosin-1, component of T7S export system	23±3	26±3	28±6	31±3	17±3	23±4	25±2	28±3	
SL363	SLIV_11935	Cytoplasmic ATP-dependent serine protease Lon	21±2	23±3	29±5	31±3	22±5	23±3	25±4	29±3	

Table S7. Primers used in this work. Primers for gene deletion are marked with the suffix “Dis”, primers used for verification of mutants phenotype are marked with suffix “Ch”. Endings “F” indicate forward and “R” reverse primers.

Primer name	Sequence (5'-3')
SLIV_10535DisF	CGCATGCCCGCACGGCATTCCTGTCGTAGCGTCCGGATATGTCGACCCGGTACCGGAGTA
SLIV_10535DisR	TTCCCGCAGAAGTAGACAGCGCGCGAGGGTGTTCACGCCTACTACGCCCCCAACTGAGAG
SLIV_20750DisF	GCCGTTGGAGGATGCAGACGGGACGTCGGCCCGCCCGTGCTCGACCCGGTACCGGAGTA
SLIV_20750DisR	GGTCGGGCCGCCGGGGCGGTCCGAGACGGCCGGGGTCAGCACTACGCCCCCAACTGAGAG
SLIV_09985DisF	TGAAGACAGCAACACGCCGAGGGCTGCGAGTACGCGTGGGTCGACCCGGTACCGGAGTA
SLIV_09985DisR	GTTGCTTCTCTCCATACGACACCGACGACGCCGGTGTCAGACTACGCCCCCAACTGAGAG
SLIV_10025DisF	ACGATCCGGAAGCGGAGGCCCGCGTGTGGCGTTGGCCGGATTCGACCCGGTACCGGAGTA
SLIV_10025DisR	TGCCCGGGCCCTGCCTCGTACCTCTGACGGCGCCGTCAGCACTACGCCCCCAACTGAGAG
SLIV_34120DisF	TGCGGAACACCGCGCGCTGGGCAGCGACTCTGGGCCTGACGTCGACCCGGTACCGGAGTA
SLIV_34120DisR	ACGCCGCACGGTCCCGGCGGTCCCGGTCCCTCAGAACGTGAAGACTACGCCCCCAACTGAGAG
SLIV_24720DisF	TGCCGGCACGCACGTGCCGGCAGCCCCCTTCGTGTGCGGTGTTCGACCCGGTACCGGAGTA
SLIV_24720DisR	CTTCACCCTGCCGACTCTTCGACATCACTTGTGGGGGCCCCACTACGCCCCCAACTGAGAG
SLIV_09410DisF	GGTAGGCGAGAGGGAAGGAAAGGACAAGCCTCTACATGGCGTCGACCCGGTACCGGAGTA
SLIV_09410DisR	GAATACAAAAAGCCCCGACTCGGGAAGCCGGGGCTTTTTCGACTACGCCCCCAACTGAGAG
SLIV_15325DisF	CAGGGACCTCCTAGGACTTCGGAGCCCCCCACATGCAGCTCTCGACCCGGTACCGGAGTA
SLIV_15325DisR	ACGTCGGCCGTCCGGTGCCCGCGCGGCGTCGCTCGCCCTACACTACGCCCCCAACTGAGAG
SLIV_31645DisF	TACTTGTCGCGAACACGTACGGGGAGGGCCACTTGAGGAAGTCGACCCGGTACCGGAGTA
SLIV_31645DisR	ACCGGTCGCCCCCTCCCCCCGCTTGGCCTGAGCCCGGGTTAACTACGCCCCCAACTGAGAG
SLIV_12485DisF	GCCGCACGGCGCGGACGGCTTGCCAGGGGGGAGAGGACATGGTCGACCCGGTACCGGAGTA
SLIV_12485DisR	GCGCACCGCAGGTGCCCGAAGCCCCCGCCGCCGTCATGAGCACTACGCCCCCAACTGAGAG
SLIV_04650DisF	GACAGCTCACCTCGCAGGCGCCGGAGAGGAATTCACCATGCTCGACCCGGTACCGGAGTA
SLIV_04650DisR	CCTTCGGTGCGCGGGGGCCGCGCGGAACCGGTCGTCAGACGACTACGCCCCCAACTGAGAG
SLIV_28740DisF	GAGCGCCGAGGCGGAGAAGGGGACGGACACCCACGATGGAATCGACCCGGTACCGGAGTA
SLIV_28740DisR	TTCGGGGCACGGGTCGCGCCCGGCGTCGACGAGTGCTGGTCACTACGCCCCCAACTGAGAG
SLIV_17030DisF	GGCGTTTCACGTGAAACACCCCATAGGATCACGAGGTGCGCTCGACCCGGTACCGGAGTA
SLIV_17030DisR	CCGCGAGGGGTGAGGCGGCGGTGGACGAGCGGGCGGCTCACGACTACGCCCCCAACTGAGAG
SLIV_11935DisF	TCGATGTAACTCAACTTGACTGCCGAAGGGGAGATCATGTCGACCCGGTACCGGAGTA
SLIV_11935DisR	ACGGGACCCGGGCCCTGCCTCTTCCGGCCGCGTCCGTCACACTACGCCCCCAACTGAGAG
SLIV_11270DisF	GGCCGCGCAACCCGTGGCCACGCAGAAGGAGTCAGTGTTGTCGACCCGGTACCGGAGTA
SLIV_11270DisR	ACTTGGGGGTGGTGCGTCCGCGGGCCGGCCGGCCTACGGGACTACGCCCCCAACTGAGAG
SLIV_11275DisF	CACGCCGTCCGGAGATCCCCCCACCGAAGGAGCTTGTGTGTCGACCCGGTACCGGAGTA
SLIV_11275DisR	CCGCCGGTCATGTCGGCCGCGGGTCAGCTCACGTTGATCGACTACGCCCCCAACTGAGAG
SLIV_02150DisF	ACGACTTCTCCCCACTCCCCCACTCAAGGAGTCATCGATGTCGACCCGGTACCGGAGTA
SLIV_02150DisR	CGGCCGGGCCGCCCGCACGGTGGTCCGCGTACGTCAGCGCACTACGCCCCCAACTGAGAG
SLIV_10535ChF	TACGTCCGCTACACCCGCAT
SLIV_10535ChR	AGCCCTACGTCTGGAAGTTC
SLIV_20750ChF	TAACGGGCTTCACGGTGTTT
SLIV_20750ChR	TCCTGCGACGGCATACGAAA
SLIV_09985ChF	CCATCATGAAGACAGCAACAC
SLIV_09985ChR	GTTGCTTCTCTCCATACGACA
SLIV_10025ChF	TGTCTGCGCGGAAGACGGAA
SLIV_10025ChR	GTGGACCCAGGAACGTGTCT
SLIV_34120ChF	TCGAAACGAGCGGAAGGATG
SLIV_34120ChR	AGTAGCGAGCAGCCGATCA
SLIV_24720ChR	CTACTTCCCCGTGAAGAGC
SLIV_24720ChR	CTACTTCCCCGTGAAGAGC
SLIV_09410ChF	AACCCGTCAGCTAACCCGG
SLIV_09410ChR	ACTCTAGGCCAATCAAGCC
SLIV_15325ChF	ATCCAGGCACCCCATTCACT
SLIV_15325ChR	GACGCCGAGGAACTGGAGT
SLIV_31645ChF	CTGTTCTCAGGAAACCCACA
SLIV_31645ChR	TACGGCGACCTCTTCGACTA
SLIV_12485ChF	CCATGGCAGGAACGCCATC
SLIV_12485ChR	GGAGAAGCCCGTCTACTGA
SLIV_04650ChF	ACCCGACAGCTCACCTCGCA
SLIV_04650ChR	AATACGGCGAGGGGCCTGTA
SLIV_28740ChF	TCTGGTCAATAGGATCTGCC

SLIV_28740ChR	AGCCGCACGTCGATACGGT
SLIV_17030ChF	TCACGTGAAACACCCCATAG
SLIV_17030ChR	CTGCTGCTGGAGACGTTCG
SLIV_11935ChF	CAATCCCGGGCTCCCTTTCA
SLIV_11935ChR	ACAACAAGTTCAACGGTGCG
SLIV_11270ChF	TCTGCGTACCGCACAGTTCC
SLIV_11270ChR	AAGGCCGGCGTAAGTGCTTG
SLIV_11275ChF	TCACCCACAGCACAACTTCG
SLIV_11275ChR	TGAAGGACATGGGCACCAAG
SLIV_02150ChF	ACACTCACCGGTGACGACTT
SLIV_02150ChR	TCTCCAGTGAACTGCGAGTA

3-Supplementary Methods

Analysis of diamide sensitivity.

Sensitivity of the *S. lividans* mutants to diamide was tested by plating spores (apr. 10^9) of each strain on fresh MS or TSB agar plates. Immediately after plating, paper discs loaded with 5 µM or 10 µM of diamide were placed, and plates were analyzed after 48 h incubation at 30°C. The tests were repeated three times for each strain. The inhibition zones were measured.

4- References

Tsolis, K.C., Tsare, E.P., Orfanoudaki, G., Busche, T., Kanaki, K., Ramakrishnan, R., et al. (2018). Comprehensive subcellular topologies of polypeptides in Streptomyces. *Microb Cell Fact* 17(1)**,** 43. doi: 10.1186/s12934-018-0892-0.
